# Emergence of *Babesia conradae* infection in coyote-hunting Greyhounds in Oklahoma, USA

**DOI:** 10.1186/s13071-021-04897-x

**Published:** 2021-08-14

**Authors:** Erin Stayton, Megan Lineberry, Jennifer Thomas, Tina Bass, Kelly Allen, Ramaswamy Chandrashekar, Gene Yost, Mason Reichard, Craig Miller

**Affiliations:** 1 Oklahoma State University, 250 McElroy Hall, Stillwater, OK USA; 2 Vinita Veterinary Center, Vinita, OK USA; 3 IDEXX Laboratories, Inc., Westbrook, ME USA; 4 Crescent Veterinary Hospital, Crescent, OK USA

**Keywords:** *Babesia conradae*, Babesiosis, Canine, Dog, Oklahoma, Greyhound, Anemia, Thrombocytopenia, *18S rRNA*

## Abstract

**Background:**

*Babesia* species are intraerythrocytic Apicomplexan parasites that infect a wide range of vertebrate hosts. These pathogens are typically transmitted either by tick vectors or by direct blood-to-blood contact, and may cause life-threatening clinical disease, such as thrombocytopenia, hemolytic anemia and acute renal failure, in canine hosts. While *Babesia vogeli* and *Babesia gibsoni* infections have both been reported in Oklahoma, reports of *Babesia conradae* infections have been limited to California.

**Methods:**

Four separate kennels of coyote-hunting dogs were identified in Oklahoma after the kennels had consulted with Oklahoma State University Boren Veterinary Medical Teaching Hospital (antemortem cases) or the Oklahoma Animal Disease Diagnostic Lab (postmortem cases). Upon owner consent, every accessible dog from each of the four kennels was briefly examined for ectoparasites, particularly ticks, and whole blood samples were collected in EDTA tubes. Clinically ill dogs were examined by a practicing veterinarian, and clinical signs included anorexia, vomiting, lethargy, fever and anemia. DNA was extracted from each blood sample, and a nested PCR was performed using general apicomplexan primers for the partial *18S rRNA* gene. PCR products were electrophoresed in agarose matrix, and appropriately sized amplicons were sequenced. Sequences were compared to reference *18S rRNA* gene sequences available in GenBank, and samples with > 98% homology to *B. conradae* (GenBank: AF158702) were considered positive. *Babesia conradae*-positive dogs were then treated with atovaquone (13.5 mg/kg three times per day) and azithromycin (10 mg/kg once daily) for 10 days and retested at 30 and 60 days post-treatment by PCR.

**Results:**

Of 40 dogs tested, 15 (37.5%) were positive for *B. conradae* with 98–99% sequence homology to *B. conradae* from California. All positive cases were coyote-hunting Greyhounds. Ectoparasites were not identified on any of the dogs at the time of blood collection. Treatment of clinically ill dogs with atovaquone and azithromycin resulted in complete clinical recovery in all treated dogs with negative follow-up PCR at 30 and 60 days post-treatment.

**Conclusions:**

Collectively, this study (i) documents the occurrence of *B. conradae* in Oklahoma, (ii) highlights this pathogen as a differential to be considered when clinical signs are present, (iii) supports the use of atovaquone and azithromycin as effective treatment in these cases and (iv) demonstrates chronic subclinical carrier dogs serving as potential reservoirs of *B. conradae* infection to naïve dogs.

**Graphical abstract:**

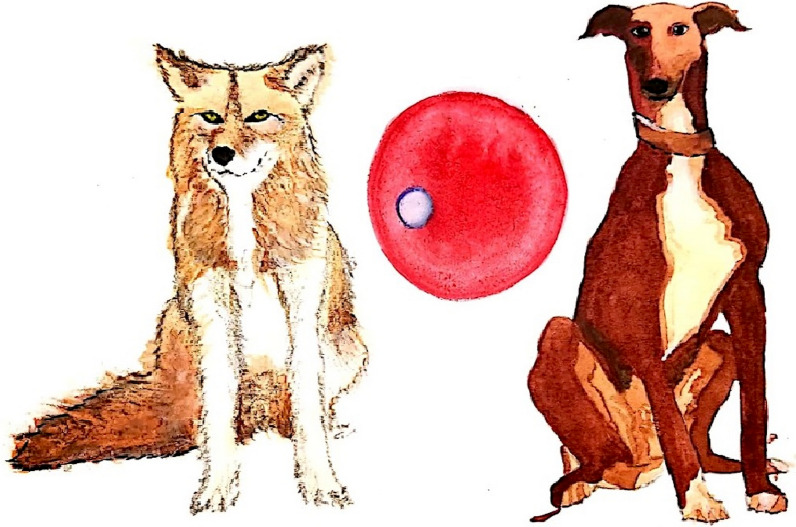

**Supplementary Information:**

The online version contains supplementary material available at 10.1186/s13071-021-04897-x.

## Background

*Babesia* species are intraerythrocytic protozoan parasites in the phylum Apicomplexa that are transmitted by the bite of an infected tick or by passage of contaminated blood to a susceptible, naïve host. There are over 100 species described, which are divided into two broad categories: small *Babesia* (measuring 1–3 µm) and large *Babesia* (measuring 3–7 µm). There are currently five named *Babesia* spp*.* enzootic to the USA known to infect dogs: *Babesia vogeli* (large), *Babesia* sp. (Coco isolate, large), *B. gibsoni* (small), *B. vulpes* (formerly *Theileria annae*, small) and *B. conradae* (small) [1–4].

*Babesia conradae* was first reported in California in 1991 as *Babesia gibsoni*, given that *B. gibsoni* was the only small *Babesia* sp. known to infect dogs at the time [5]. Further characterization of the piroplasm in 2006 revealed that the California organism was a distinct species, and the name was subsequently changed to *B. conradae* after the first reporting author, Dr. Patricia Conrad [6]. The transmission dynamics of *B. conradae* remain unknown as attempts at tick transmission of this piroplasm have not been successful [7].

Dogs infected with *B. conradae* exhibit typical clinical signs of babesiosis, including anorexia, hemolytic anemia, splenomegaly, thrombocytopenia and vomiting [5]. Similar to infections caused by other canine *Babesia* spp., clinical signs resulting from *B. conradae* infection can vary and range from mild to life-threatening. Severe complications, such as acute renal failure/renal disease, cardiac related alterations, acute respiratory distress syndrome and acute pancreatitis, may result, particularly in dogs with *B. conradae* infection [2, 5]. Membranoproliferative glomerulonephritis has been reported in one patient. Case fatality rate in *B. conradae*-infected patients can reach up to 40% without timely and appropriate therapeutic intervention [5]. While various medications have been attempted, combination therapy with atovaquone and azithromycin is the only treatment regimen shown to successfully clear *B. conradae* infection in dogs [8].

*Babesia vogeli* and *B. gibsoni* infections have been previously reported throughout the USA and in the state of Oklahoma; however, *B. conradae* infection has not yet been documented outside of the state of California. In the current study, we surveyed kenneled coyote-hunting dogs for *B. conradae* and other apicomplexan parasites in Oklahoma and evaluated the efficacy of atovaquone and azithromycin therapy [8, 9] in treating dogs with clinical disease.

## Methods

### Study population

Four separate kennels (groups) of coyote-hunting dogs were identified following presentation of severe acute illness in at least one dog in the kennel or in a closely associated kennel. Groups 1, 3 and 4 sought consultation at either the Oklahoma State University Boren Veterinary Medical Teaching Hospital/College of Veterinary Medicine (antemortem cases) or the Oklahoma Animal Disease Diagnostic Laboratory (postmortem cases). Group 2 volunteered for testing given their working association with Group 1. All kenneled dogs in each group were included in the study. All kennels were located in Oklahoma.

**Fig. 1:**
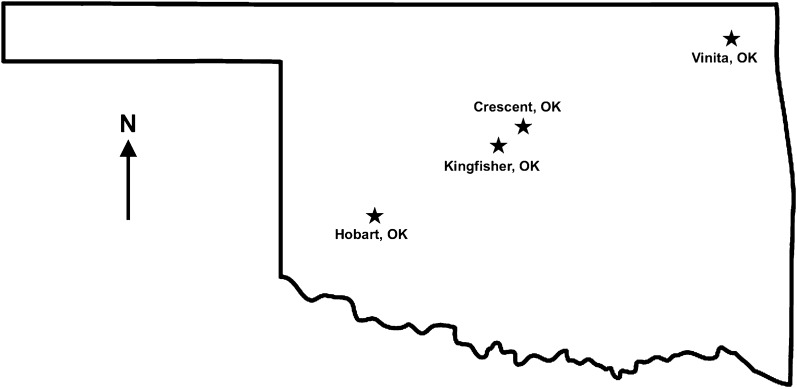
Geographical distribution of *Babesia conradae* cases in Oklahoma. A total of 40 dogs were included in this study from four separate kennels in Oklahoma: 6 Greyhounds and 4 Treeing Walker Coonhounds from Crescent, OK (Group 1), 16 Greyhounds from Kingfisher, OK (Group 2), 10 Greyhounds from Vinita, OK (Group 3) and 4 Greyhounds from Hobart, OK (Group 4)

Group 1 consisted of six Greyhounds and four Treeing Walker Coonhounds from a kennel in Crescent, OK, sampled in March 2014; Group 2 consisted of 16 Greyhounds from a kennel in Kingfisher, OK, also sampled in March of 2014; Group 3 consisted of ten Greyhounds from a kennel in Vinita, OK, sampled in February 2019; and Group 4 consisted of four Greyhounds from a kennel in Hobart, OK, sampled in May 2020 (Fig. 1). One dog from Group 1 displayed clinical signs of lethargy, fever and anemia (Dog 1) while the remaining dogs were subclinical (bright, alert, responsive, good body condition, good appetite, hunted effectively and had no apparent clinical signs, such as fever/lethargy). Clinical signs were not apparent in any of the dogs in Group 2. In Group 3, two dogs (Dogs 27 and 28) showed clinical signs, including vomiting, lethargy, anorexia and anemia, while the remaining dogs were subclinical. Group 4 had one dog showing clinical signs, including profound lethargy, anorexia, icterus and splenomegaly, which died prior to definitive diagnosis and treatment (Dog 37) and one subclinical dog.

Housing varied from kennel to kennel. Group 1 dogs were housed in individual horse stalls, dogs in Groups 2 and 4 were kept in outdoor chain link runs with dirt bedding and dogs in Group 3 were in outdoor wood panel runs on a combination of dirt and gravel. At initial presentation, each dog was briefly examined for ectoparasites, particularly ticks. Patient temperament, however, impeded an extensive dermatological evaluation. For tick prevention, Group 1 received Certifect (Merial, Duluth, GA, USA) monthly, Groups 2 and 3 were not on tick prevention and Group 4 received Simparica (Zoetis, Kalamazoo, MI, USA), treatment duration unknown. Whole blood (1–5 ml) was collected in EDTA tubes from all animals (*n* = 40). Blood was transported on ice to the Oklahoma State University College of Veterinary Medicine (OSU-CVM) and stored at 4 °C prior to processing.

### Serologic testing and blood smear evaluation

All Group 3 samples were tested using a standard SNAP® 4Dx® Plus test according to manufacturer’s instructions (IDEXX, Westbrook, Maine, USA). Blood smears were prepared for Group 1 samples upon arrival to Oklahoma State University using a previously published method and evaluated microscopically [10]. Blood samples for blood smear evaluation were not available for Groups 2–4 and were therefore not evaluated.

### DNA extraction and PCR

For Groups 1 and 2, DNA was extracted from whole blood using the DNeasy Blood and Tissue Kit (Qiagen, Valencia, CA, USA). For Groups 3 and 4, DNA was extracted using the Illustra™ blood genomic Prep Mini Spin Kit (GE Healthcare, Piscataway, New Jersey, USA). All extractions were performed according to the manufacturers’ instructions. Dedicated laboratory areas were utilized for DNA extractions, primary and secondary PCR amplifications and PCR product purifications to prevent contamination events. Separate ultra-purified water samples (no template control [NTC]) were included as negative controls in DNA extractions and PCR amplifications. DNA extracts from Groups 1–4 were analyzed by previously described nested PCR methods which amplify a 460- to 520-bp hypervariable region of the *18S rRNA* gene of *Babesia* spp. and other apicomplexans (*Hepatozoon* spp., *Sarcocystis* spp. and *Toxoplasma* spp.) (Table 1) [11, 12], except for Dog 27 for which the PCR and sequencing was carried out by the North Carolina State Vector Borne Disease Diagnostics Lab (NCSVBDDL) per their standard operating procedures. NCSVBDDL utilizes primers that also amplify the *18S rRNA* gene. DNA extracts from known piroplasm-positive blood samples by microscopy were included as positive controls for each sample set. Dogs in Group 3 with antibodies to tick-borne bacteria (namely *Ehrlichia* spp.) as detected by the SNAP 4Dx assay were tested by PCR to potentially amplify and identify circulating organism as previously described [13].

**Table 1:** PCR primers used to amplify partial *18S rRNA* gene fragments of *Babesia* spp. and other apicomplexans

Primer	Primer sequence (5′→ 3′)	Reference
BABA-F	CCGAATTCGACAACCTGGTTGATCCTGCCAGT	[11]
BABA-R	CCCGGATCCAAGCTTGATCCTTCTGCAGGTTCACCTAC	[11]
3.1	CTCCTTCCTTTAAGTGATAAG	[12]
5.1	CCTGGTTGATCCTGCCAGTAGT	[12]
RLB-F	GAGGTAGTGACAAGAAATAACAATA	[12]
RLB-R	TCTTCGATCCCCTAACTTTC	[12]

For Groups 1 and 2, primary PCR reactions were performed in 25-µl volumes containing 0.25 U Taq polymerase (Promega, Madison, WI, USA), 10× Taq buffer (Promega), 1.5 mM MgCl_2_, 0.8 mM dNTP mixture (Promega), 0.8 µM each primer BABA-F and BABA-R and 5 µl template DNA. Primary reaction conditions were as follows: 94 °C, 5 min; then 94 °C/1 min, 56.6 °C/1 min, 72 °C/2 min for 35 cycles; and a final extension step of 72 °C for 5 min. Nested PCR was carried out using 1 µl of the primary product and primers RLB-F and RLB-R. Nested reaction conditions were as follows: 94 °C, 5 min; then 94 °C/1 min, 50 °C/1 min, 72 °C/2 min for 35 cycles; and a final extension step of 72 °C for 5 min.

For Groups 3 and 4, primary PCR reactions were prepared in 25-µl volumes containing 0.075 U Accuprime™ Taq HIFI (Thermo Fisher Scientific, Waltham, MA, USA), 1× AccuPrime™ PCR Buffer II (Thermo Fisher Scientific), 1.5 mM MgSO_4_ (ThermoFisher), 0.2 µM each primer 3.1 and 5.1, and 5 µl of DNA extract. Primary reaction conditions were as follows: 94 °C for 2 min followed by 30 cycles of 94 °C for 1 min, 55 °C for 1 min, 68 °C for 1.5 min, and a final extension step of 72 °C for 10 min. Nested PCR was again carried out using 1 µl of primary product and primers RLB-F and RLB-R, but cycling conditions were different than above. For Group 3 and 4 dogs, nested PCR reaction conditions were as follows: 94 °C, 2 min; then 94 °C/1 min, 50 °C/1 min, 68 °C/1.5 min for 40 cycles; and a final extension step of 72 °C for 10 min.

### PCR product purification and sequencing

PCR products were electrophoresed in a 2% agarose matrix containing either GelRed® QIAquick® Gel Extraction Kit (Qiagen) or the Wizard® SV Gel and PCR Clean-Up System (Promega) according to manufacturers’ instructions. DNA sequencing was performed at the Oklahoma State University Molecular Core Facility (Stillwater, OK, USA) with an ABI 3730 DNA Analyzer (Applied Biosystems, Thermo Fisher Scientific), except for Dog 27. Forward and reverse sequences were aligned with ClustalW (Bioinformatics Center, Kyoto, Japan) and compared with sequence data available in the National Center for Biotechnology Information database (GenBank) for *B. conradae* (AF158702), *B. gibsoni* (AF205636) and *B. vogeli* (AY371198) to determine percent homology. Samples were considered positive if they were ≥ 98% homologous to *B. conradae* (GenBank: AF158702). The sequences from Dogs 1, 28, 29, 33, 34, 35, 37 and 38 have been deposited in GenBank under the accession numbers MW147022, MT430944, MW145168, MW145196, MW145199, MW145504, MW145505 and MW145506, respectively.

### Phylogenetic tree and percent identity matrix construction

All partial *18S rRNA* gene sequences obtained from canines in the current study were entered into MacVector, then aligned and trimmed along with sequences from various piroplasm reference sequences available in GenBank which were used in previous analyses [1, 6, 14]. A maximum likelihood phylogenetic tree was constructed using unweighted pair group method with arithmetic mean (UPGMA) analysis. Bootstrap values are based on 1000 replicates and only bootstraps > 50% are indicated. The percent homology matrix was also produced in MacVector using the same *18S rRNA* gene full and partial sequences.

### Treatment protocol

Upon owner consent, surviving *B. conradae* PCR-positive dogs from each cohort were treated with a previously described treatment regimen shown to eliminate infection* in vivo* [8], except for Dog 4 which was sold and lost to follow-up. Atovaquone (GlaxoSmithKline, Research Triangle Park, NC, USA) and azithromycin (Pfizer, New York, NY, USA) were compounded in an oral suspension and administered to all surviving PCR-positive dogs at a dose of 13.5 mg/kg TID (atovaquone) and 10 mg/kg SID (azithromycin) for 10 days. Whole blood (1–5 ml) was collected in EDTA tubes prior to treatment (day 0) and at 30 and 60 days post-treatment. DNA was extracted from each blood sample and tested by PCR to detect *Babesia* sp. infection as previously described.

### Statistics

The prevalence of *B. conradae* infection was calculated according to Bush et al. [15] and 95% confidence intervals (CIs) were calculated using QuickCalcs GraphPad (https://www.graphpad.com/quickcalcs/). The prevalence of *B. conradae* in hunting dogs among kennel age groups were compared using chi-square (*X*^2^)  tests [16].

## Results

### PCR analysis of dogs screened for *B. conradae* infection

From the 40 coyote-hunting dogs screened for *B. conradae* infection, 15 (37.5%; 95% CI 24.1–53.0%) were positive by PCR and sequencing for *B. conradae* infection (Additional file 1: Table S1). This included three of ten (30.0%; 95% CI 10.3‒60.8%) dogs from Group 1 (Crescent, OK; all Greyhounds), four of 16 (25%; 95% CI 9.7‒50.0%) dogs from Group 2 (Kingfisher, OK; all Greyhounds), six of ten (60.0%; 95% CI 31.2‒83.3%) dogs from Group 3 (Vinita, OK; all Greyhounds) and two of four (50.0%; 95% CI 15.0–85.0%) dogs from Group 4 (Hobart, OK; all Greyhounds). A significant difference in the prevalence of *B. conradae* among the kennels was not detected (*X*^2^ = 3.733, *df* = 2, *P* = 0.292).

PCR-positive dogs ranged in age from 1.5–6 years ($\overline{x }$ = 3.7 years; 95% CI 2.6–4.8), with age unknown in one positive dog. PCR-negative dogs ranged in age from 5 months to 10 years ($\overline{x }$ = 2.8 years; 95% CI 1.68–3.9). There was no significant difference in age groups of PCR-positive and -negative dogs (*X*^2^ = 3.911, *df* = 3, *P* = 0.271). Of the PCR-positive dogs in Group 3, four were females and two were males. Of the PCR-positive dogs in Group 4, both were males. *Hepatozoon canis* and *Ehrlichia canis* were confirmed based on sequence in dogs 11 and 31, respectively.

### SNAP® 4Dx® Plus results

Of the dogs tested by SNAP® 4Dx® Plus test (Group 3, *n* = 10), only Dog 31 had antibodies to *Ehrlichia* spp. All other Group 3 dogs were negative for *Anaplasma* spp., *Dirofilaria immitis*, *Ehrlichia* spp. and *Borrelia burgdorferi* antibodies by the SNAP® 4Dx® Plus test.

### Clinicopathologic findings

**Fig. 2:**
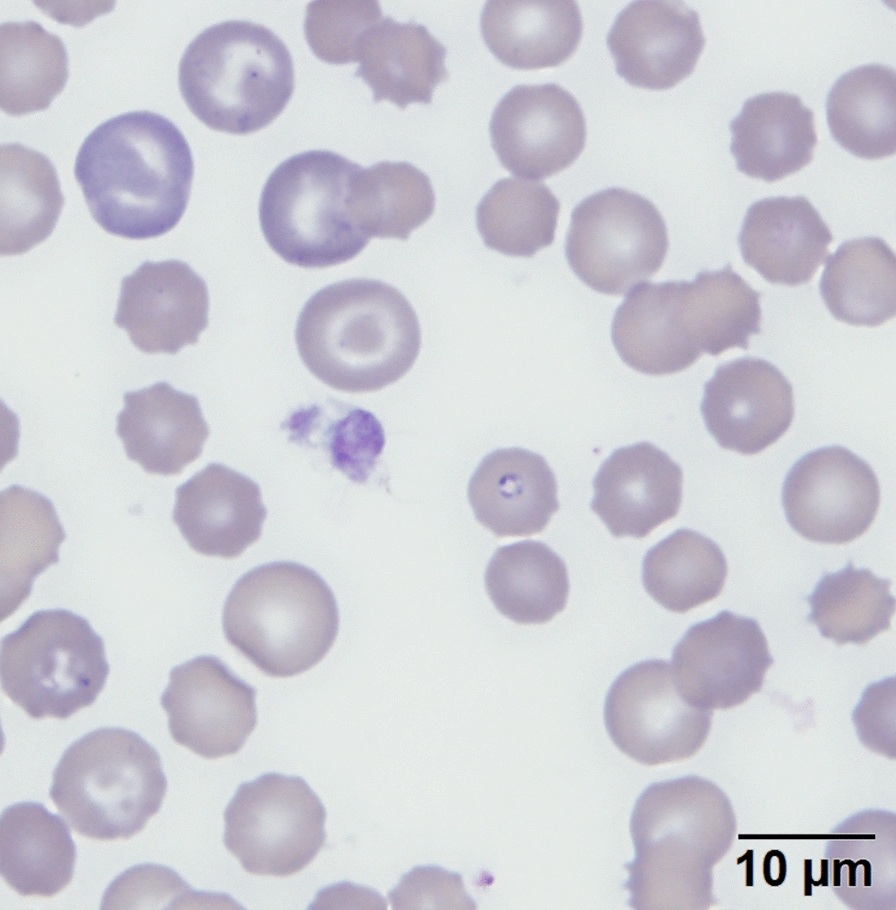
Blood smear from a *Babesia conradae*-positive Greyhound in Group 1. Approximately 10% of the erythrocytes contain pale basophilic intracellular protozoa, consistent with intraerythrocytic *B. conradae* piroplasms. A mild degree of polychromasia is also present within the specimen, indicating regenerative anemia. Scale bar: 10 µm

Clinicopathologic data (serum biochemistry, complete blood counts) were available for two clinically ill dogs who survived infection (Dogs 1 and 27), with the clinical signs of regenerative anemia, thrombocytopenia, hyperglycemia and hypocalcemia in both animals. One dog from the same kennel as Group 3 dogs exhibited the aforementioned changes as well as severe azotemia. This dog passed away prior to diagnosis and was the only dog submitted for postmortem examination. We were unable to obtain a sequence on this dog, and it is therefore not included in the total sample set; however, postmortem microscopic lesions included membranoproliferative glomerulonephritis, tubular proteinosis, myocardial necrosis and necrosuppurative opportunistic bronchopneumonia that cultured *Escherichia coli*, *Staphylococcus pseudintermedius* and *Streptococcus minor*. The renal and myocardial lesions have both been reported in canine Babesiosis [2]. Evaluation of blood smears of infected dogs from Group 1 revealed mild polychromasia (indicative of regenerative anemia) and numerous basophilic, intraerythrocytic piroplasms consistent with *Babesia* sp. (Fig. 2). Blood smears were not evaluated for dogs from Groups 2–4.

### Sequence analysis

**Fig. 3:**
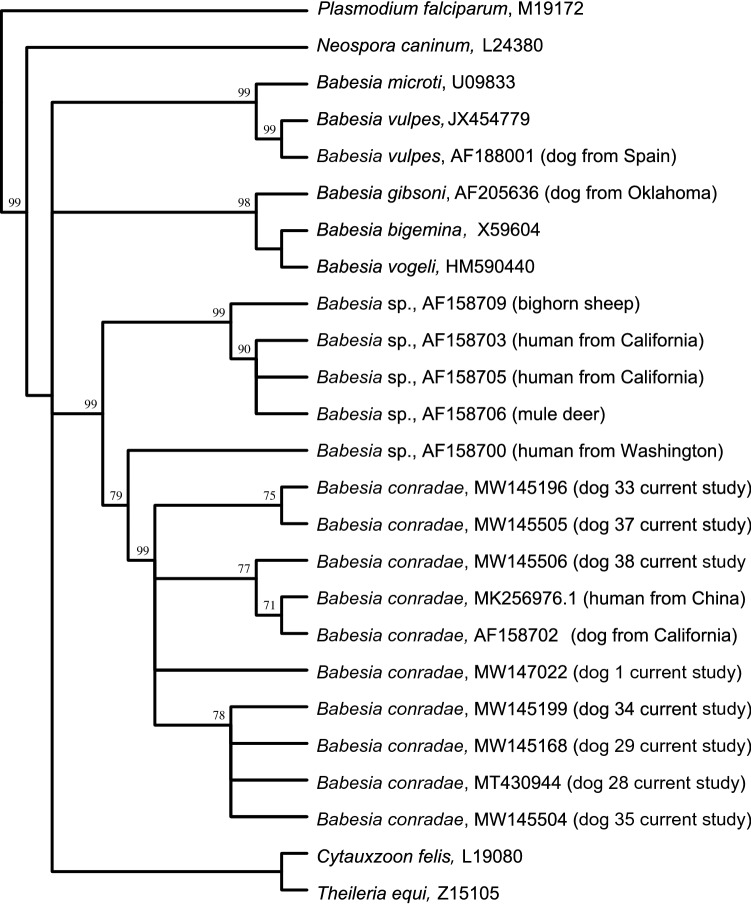
Phylogenetic comparison of *Babesia conradae* isolates from Oklahoma to other piroplasms. Maximum likelihood phylogenetic tree constructed using unweighted pair group method with arithmetic mean analysis of *18S rRNA* gene sequences extracted from GenBank. Clustering of study samples within a distinct subclade (red lines) illustrates the homology of these Oklahoma strains to the original (California) *B. conradae* strains (GenBank: MK256976 and AF158702) and highlights the distinct separation of study samples from other *Babesia* spp. Bootstrap values are based on 1000 replicates and only bootstraps < 50% are indicated

The *18S rRNA* gene sequences exhibited 98–99% homology to *B. conradae* (GenBank: AF158702) and 97–100% homology with each other, and shared 78% homology with *B. gibsoni* and *B. vogeli* (Table 2). Phylogenetic analysis demonstrated a distinct relationship between the Oklahoma *Babesia* sp. and the California *B. conradae* strains; sequences from Oklahoma dogs clustered with *B. conradae* sequences documented from California dogs (GenBank: MK256976 and AF158702) and were more distant to other *Babesia* spp. sequences used in the comparison (Fig. 3).

**Table 2:** Percent identity matrix of Oklahoma *Babesia conradae 18S rRNA* gene sequences compared to reference sequences in GenBank

Dog ID	Dog 1	Dog 27	Dog 28	Dog 29	Dog 33	Dog 34	Dog 35	Dog 37	Dog 38	*Babesia conradae* (AF158702)	*Babesia gibsoni* (AF205636)	*Babesia vogeli* (AY371198)
Dog 1	100	98	99	99	98	98	98	98	98	98	78	78
Dog 27	98	100	98	98	98	97	98	98	98	98	78	78
Dog 28	99	98	100	100	98	99	99	98	99	99	78	78
Dog 29	99	98	100	100	98	99	99	98	97	99	78	78
Dog 33	98	98	98	98	100	98	98	99	98	98	78	78
Dog 34	98	97	99	99	98	100	99	98	98	98	78	78
Dog 35	98	98	99	99	98	99	100	98	98	99	78	78
Dog 37	98	98	98	98	99	98	98	100	98	98	78	78
Dog 38	98	98	99	99	97	98	98	98	100	99	78	78
*Babesia conradae* (AF158702)	98	98	99	99	98	98	99	98	99	100	79	78
*Babesia gibsoni* (AF205636)	78	78	78	78	78	78	78	78	78	79	100	89
*Babesia vogeli* (AY371198)	78	78	78	78	78	78	78	78	78	78	89	100

GenBank accession numbers are given in parentheses

### Response to therapy

Of the PCR-positive dogs in Groups 1–4, 13 dogs (Additional file 1: Table S1) were administered a combination therapy of atovaquone and azithromycin for 10 days as previously described and then re-tested by PCR at 30 and 60 days post-treatment. In one case (Dog 27) three blood transfusions were required to stabilize the patient during treatment. Dog 4 was sold and lost to follow-up. Dog 37 passed away shortly after blood was collected for testing and was therefore not in the treatment group. Complete resolution of clinical signs was observed in all sick dogs by day 4 of treatment, and *B. conradae* DNA was not detected in the blood of any dog at 30 or 60 days post-treatment.

Case follow-up was conducted* via* telephone conversation with the owners approximately 1 year post-treatment. Previously subclinical dogs reportedly continued to do well with no change noted by the owners. Clinically ill dogs recovered well, gained weight back that had been lost and returned to hunting the following season. Dog 38 (Group 4, subclinical) notably improved in body condition, energy level and coat quality as noted by the first and second authors at the 60 day blood collection.

## Discussion

Multiple outbreaks of *B. conradae* have been previously reported in dogs from southern California since 1991 [5, 8, 17, 18], but infection has not yet been reported outside this core, initial area. A single report of *B. gibsoni*-like parasite genetically similar to *B. conradae* was documented in Oklahoma in 2001 (GenBank: AF205636) [19], but further BLAST analysis by the authors showed 100% alignment with numerous *B. gibsoni* sequences and significant genetic divergence from *B. conradae* (76.8% homologous to *B. conradae*, GenBank: AF158702). Full travel and family histories are unavailable for all dogs in the current study; however, none of the dogs originated from or had been transported to California. To the authors’ knowledge, this is the first published report of *B. conradae* infection outside of California and represents the emergence of an important pathogen in Oklahoma that is capable of causing significant disease in kenneled dogs.

Diagnosis of babesiosis is typically based on clinical signs and/or observation of intraerythrocytic piroplasms on blood smear (Fig. 2). Ancillary testing (PCR and DNA sequencing) is required for accurate identification given that small piroplasms are microscopically indistinguishable from each other, both those within the *Babesia* genus and those from *Theileria* spp., and there is cross reactivity with immunofluorescence antibody (IFA) testing [20]. Additionally, *B. conradae* parasitemia can be low (< 1% of erythrocytes with piroplasms), making detection of the organism on blood smear difficult. PCR has become widely available and is both sensitive and specific for detection of *B. conradae* DNA using the *18S rRNA* gene [21]. Differentiation between the small *Babesia* spp. is important, given that *B. conradae* can be more pathogenic than *B. gibsoni* [5, 8, 22].

Testing for disease and identification of the disease have previously and primarily been conducted in clinically ill dogs. However, in the current study, we observed four *B. conradae*-positive dogs with clinical babesiosis and ten with subclinical infections. Our observation of ten subclinical dogs suggests that some dogs may be chronic carriers and possibly serve as a reservoir of infection for naïve animals. Further research should determine if *B. conradae* is more prevalent in high-risk groups than previously thought. It remains to be determined if there are any negative long-term health problems associated with a chronic carrier state and whether atovaquone and azithromycin treatment would be beneficial in these animals.

*Babesia* spp. are primarily transmitted by ticks worldwide; however, transmission mechanisms of *Babesia* spp. in the USA differ. For example, *B. vogeli* is transmitted by *Rhipicephalus sanguineus* (*s.l.*) (brown dog tick), while *B. gibsoni* is largely considered to be transmitted by passage of contaminated blood during dog fights [5]. Comparatively, the source of *B. conradae* infection in domestic dogs is still unknown, and it is unclear whether transmission occurs* via* a tick vector, by transfer of contaminated blood between dogs, trans-placentally from an infected dam or by a previously undocumented route [7].

There is some evidence that other *Babesia* spp. can be transmitted trans-placentally, but for this study a detailed family history was not available to assess the possibility for vertical *B. conradae* infection in these Greyhounds [23]. Kennel owners in the present study suspected that their dogs were becoming infected with *B. conradae* by fighting coyotes while hunting. A previous report of *B. conradae* infection in coyote-hunting dogs in southern California also documented that infection was associated with a history of aggressive interactions with coyotes [17]. A serosurvey performed in California in 1994 showed that three of nine coyotes were seropositive for *Babesia gibsoni*, suggesting these wild canids may be a reservoir host for *Babesia* sp. [18]. At that time *B. conradae* had not yet been recognized, highlighting the potential for these seropositive coyotes to have been actually infected with multiple *Babesia* spp., including *B. conradae*. Moreover, these studies used IFA for organism detection, which is also subject to cross-reactivity [20]. It is also possible to have a subclinical carrier dog transmit the disease to a naïve dog during hunting, as the dogs may bite each other during the interaction with the coyote. Although speculative at this time, all of these scenarios represent possible reservoirs for *B. conradae* infection in these study animals, and studies are currently underway to investigate the presence of *B. conradae* in wild tick populations, free-ranging coyotes and other domestic canids* via* contact tracing to determine the source of this pathogen in the Oklahoma region.

Some *Babesia* spp. can infect multiple vertebrate species, such as *B. microti*, which has been identified in both rodents and humans, and *B. divergens*, identified in both cattle and gerbils [21]. Phylogenetic analyses showed *B. conradae* to be closely related to piroplasms from humans, bighorn sheep and mule deer [1]. Determining the suitability of various hosts for *B. conradae*, particularly humans, is an important area needing further research, especially when the route of transmission remains undetermined. A maximum likelihood phylogenetic tree (Fig. 3) constructed from samples with sequence data and other sequences available in GenBank demonstrates a close relationship of the *B. conradae* strains from Oklahoma with those from California. It also demonstrates a clear distinction from *B. gibsoni* [1, 14].

*Babesia conradae* infection in these animals resulted in a variety of clinical signs, including fever, vomiting, anorexia, regenerative anemia, and thrombocytopenia—all of which were mitigated by treatment with atovaquone and azithromycin. The source of *B. conradae* infection in Oklahoma remains unknown. At this time, *B. conradae* infection in Oklahoma has been documented in only Greyhounds used for hunting coyotes.

## Conclusions

In conclusion, the current study documents the emergence of *B. conradae* infection in 15 coyote-hunting Greyhounds in Oklahoma. Treatment with atovaquone and azithromycin promoted the mitigation of clinical signs and also reduced *B. conradae* DNA to undetectable levels. Further research is needed to determine the regional prevalence, reservoir host(s), mode(s) of transmission and diversity of vertebrate host species. Similarly, surveillance should be expanded to include other areas of the Midwest and Southern geographical regions of the USA.

## Supplementary Information

**Additional file 1: Table S1.** PCR results of coyote hunting dogs in Oklahoma tested for infection with *Babesia conradae*.

## Data Availability

The sequences from dogs 1, 28, 29, 33, 34, 35, 37 and 38 have been deposited in GenBank under the accession numbers MW147022, MT430944, MW145168, MW145196, MW145199, MW145504, MW145505 and MW145506, respectively.

## Abbreviations

EDTA: Ethylenediaminetetraacetic acid
rRNA: Ribosomal ribonucleic acid
SID: Once daily
TID: Three times daily
