# Supplementary material for: Emergence of Babesia conradae infection in coyote-hunting Greyhounds in Oklahoma, USA
Source: Parasit Vectors. 2021 Aug 14;14:402. doi: 10.1186/s13071-021-04897-x (PMC8364010; doi:10.1186/s13071-021-04897-x)
Supplement: Supplementary file 1 — Additional file 1: Table S1. PCR results of coyote hunting dogs in Oklahoma tested for infection with Babesia conradae. [file 13071_2021_4897_MOESM1_ESM.docx]

**Additional file 1: Table S1.** PCR results of coyote hunting dogs in Oklahoma tested for infection with *Babesia conradae*. Dogs treated with atovaquone & azithromycin therapy are indicated in bold (see text for details).

| Dog number | Age in years | Gender | Breed | Group | Location | Day 0 | Day 30 | Day 60 |
| --- | --- | --- | --- | --- | --- | --- | --- | --- |
| 1 | **4** | **NA** | **Greyhound** | **1** | **Crescent, OK** | **+** | **-** | **-** |
| 2 | 2 | NA | Greyhound | 1 | Crescent, OK | - | NA | NA |
| 3 | **4** | **NA** | **Greyhound** | **1** | **Crescent, OK** | **+** | **-** | **-** |
| 4 | **2** | **NA** | **Greyhound** | **1** | **Crescent, OK** | **+** | **NA** | **NA** |
| 5 | 3 | NA | Greyhound | 1 | Crescent, OK | - | NA | NA |
| 6 | 0.75 | NA | Greyhound | 1 | Crescent, OK | - | NA | NA |
| 7 | 6 | NA | Treeing walker coonhound | 1 | Crescent, OK | - | NA | NA |
| 8 | 5 | NA | Treeing walker coonhound | 1 | Crescent, OK | - | NA | NA |
| 9 | 4 | NA | Treeing walker coonhound | 1 | Crescent, OK | - | NA | NA |
| 10 | 0.75 | NA | Treeing walker coonhound | 1 | Crescent, OK | - | NA | NA |
| 11 | **1.5** | **NA** | **Greyhound** | **2** | **Kingfisher, OK** | **+** | **-** | **-** |
| 12 | 5 | NA | Greyhound | 2 | Kingfisher, OK | - | NA | NA |
| 13 | **NA** | **NA** | **Greyhound** | **2** | **Kingfisher, OK** | **+** | **-** | **-** |
| 14 | 6 | NA | Greyhound | 2 | Kingfisher, OK | - | NA | NA |
| 15 | 3 | NA | Greyhound | 2 | Kingfisher, OK | - | NA | NA |
| 16 | 1.6 | NA | Greyhound | 2 | Kingfisher, OK | - | NA | NA |
| 17 | 1.6 | NA | Greyhound | 2 | Kingfisher, OK | - | NA | NA |
| 18 | 0.4 | NA | Greyhound | 2 | Kingfisher, OK | - | NA | NA |
| 19 | **1.5** | **NA** | **Greyhound** | **2** | **Kingfisher, OK** | **+** | **-** | **-** |
| 20 | **1.5** | **NA** | **Greyhound** | **2** | **Kingfisher, OK** | **+** | **-** | **-** |
| 21 | 10 | NA | Greyhound | 2 | Kingfisher, OK | - | NA | NA |
| 22 | 0.4 | NA | Greyhound | 2 | Kingfisher, OK | - | NA | NA |
| 23 | 0.4 | NA | Greyhound | 2 | Kingfisher, OK | - | NA | NA |
| 24 | 7 | NA | Greyhound | 2 | Kingfisher, OK | - | NA | NA |
| 25 | 0.5 | NA | Greyhound | 2 | Kingfisher, OK | - | NA | NA |
| 26 | 7 | NA | Greyhound | 2 | Kingfisher, OK | - | NA | NA |
| 27 | **3** | **F** | **Greyhound** | **3** | **Vinita, OK** | **+** | **-** | **-** |
| 28 | **5** | **F** | **Greyhound** | **3** | **Vinita, OK** | **+** | **-** | **-** |
| 29 | **6** | **M** | **Greyhound** | **3** | **Vinita, OK** | **+** | **-** | **-** |
| 30 | 1 | F | Greyhound | 3 | Vinita, OK | - | NA | NA |
| 31 | 0.9 | M | Greyhound | 3 | Vinita, OK | - | NA | NA |
| 32 | 1 | F | Greyhound | 3 | Vinita, OK | - | NA | NA |
| 33 | **5** | **F** | **Greyhound** | **3** | **Vinita, OK** | **+** | **-** | **-** |
| 34 | **7** | **F** | **Greyhound** | **3** | **Vinita, OK** | **+** | **-** | **-** |
| 35 | **2** | **M** | **Greyhound** | **3** | **Vinita, OK** | **+** | **-** | **-** |
| 36 | 1 | M | Greyhound | 3 | Vinita, OK | - | NA | NA |
| 37 | 6 | M | Greyhound | 4 | Hobart, OK | + | Deceased | Deceased |
| 38 | **3** | **M** | **Greyhound** | **4** | **Hobart, OK** | **+** | **-** | **-** |
| 39 | 0.75 | M | Greyhound | 4 | Hobart, OK | - | NA | NA |
| 40 | 0.74 | M | Greyhound | 4 | Hobart, OK | - | NA | NA |

Abbreviations: F, female; M, male, NA, not applicable/available; OK, Oklahoma.
